# Testing for Antibodies Against Aquaporin-4 and Myelin Oligodendrocyte Glycoprotein in the Diagnosis of Patients With Suspected Autoimmune Myelopathy

**DOI:** 10.3389/fneur.2022.912050

**Published:** 2022-05-20

**Authors:** Samir Alkabie, Adrian Budhram

**Affiliations:** ^1^Department of Clinical Neurological Sciences, London Health Sciences Centre, Western University, London, ON, Canada; ^2^Deparment of Pathology and Laboratory Medicine, London Health Sciences Centre, Western University, London, ON, Canada

**Keywords:** myelitis, autoimmune neurology, neuroimmunology, neuroinflammation, autoantibody

## Abstract

Autoimmune myelopathies are immune-mediated disorders of the spinal cord that can cause significant neurologic disability. Discoveries of antibodies targeting aquaporin-4 (AQP4-IgG) and myelin oligodendrocyte glycoprotein (MOG-IgG) have facilitated the diagnosis of autoimmune myelopathies that were previously considered to be atypical presentations of multiple sclerosis (MS) or idiopathic, and represent major advancements in the field of autoimmune neurology. The detection of these antibodies can substantially impact patient diagnosis and management, and increasing awareness of this has led to a dramatic increase in testing for these antibodies among patients with suspected autoimmune myelopathy. In this review we discuss test methodologies used to detect these antibodies, the role of serum vs. cerebrospinal fluid testing, and the value of antibody titers when interpreting results, with the aim of helping laboratorians and clinicians navigate this testing when ordered as part of the diagnostic evaluation for suspected autoimmune myelopathy.

## Introduction

Autoimmune myelopathies are immune-mediated disorders of the spinal cord that can cause profound weakness, numbness, and bowel/bladder dysfunction. Antibodies targeting aquaporin-4 (AQP4-IgG) and myelin oligodendrocyte glycoprotein (MOG-IgG) define disease processes that can cause immune-mediated spinal cord dysfunction and have clinical features, treatment considerations and prognoses distinct from multiple sclerosis (MS) (1–17). Recognition of their substantial impact on patient diagnosis and management has led to a dramatic increase in testing for these antibodies among patients with suspected autoimmune myelopathy, which parallels an increase in neural antibody testing for suspected neurological autoimmunity more generally (18–20). We herein discuss test methodologies used to detect these antibodies, the role of serum vs. cerebrospinal fluid (CSF) testing, and the value of antibody titers in diagnostic interpretation of results. Key testing considerations for these antibodies are summarized in Table 1. Other aspects of these antibodies separate from their diagnostic utility in the clinical evaluation of suspected autoimmune myelopathy, such as how their titers relate to disease severity, how their persistent positivity informs risk of relapse, or how they interact with the complement system, are not the focus of this review but have been studied and discussed elsewhere (15, 21–26).

**Table 1:** Key considerations when testing for antibodies against aquaporin-4 (AQP4) and myelin oligodendrocyte glycoprotein (MOG).

**Target antigen**	**Typical clinical presentations that should prompt consideration of testing^1^**	**Preferred test methodology**	**Preferred test specimen**	**Practical considerations of testing performed in clinical practice**
AQP4	Optic neuritis, myelitis, area postrema syndrome, other brainstem syndrome, symptomatic narcolepsy/diencephalic syndrome with typical brain MRI lesions, symptomatic cerebral syndrome with typical brain MRI lesions	CBA	Serum is preferred because sensitivity is higher than CSF; CSF typically only positive in patients with high serum titers	Fixed and live CBA have been reported to have comparably high sensitivity and specificity; ELISA is still in clinical use, but CBA is preferred due to superior diagnostic performance
MOG	Optic neuritis, acute disseminated encephalomyelitis, myelitis, brainstem syndrome, unilateral cerebral cortical encephalitis	CBA	Serum is preferred because overall sensitivity is higher than CSF; isolated CSF positivity rarely reported and would benefit from further study	Live CBA reported to confer some diagnostic advantage over fixed CBA that would benefit from further study; low antibody titers should be interpreted with caution in patients with atypical presentations

1 *Presumes acute/subacute presentation of otherwise unknown etiology. CBA, cell-based assay; CSF, cerebrospinal fluid; ELISA, enzyme-linked immunosorbent assay; MRI, magnetic resonance imaging*.

## Antibodies Against Aquaporin-4

### “NMO-IgG”: A Novel Disease Biomarker Discovered by Tissue Indirect Immunofluorescence

Neuromyelitis optica (NMO), now termed neuromyelitis optica spectrum disorders (NMOSD) and historically known as Devic's syndrome, is a neuro-inflammatory disease that classically presents with relapsing optic neuritis and myelitis (27). In 2004, a serum IgG that characteristically stained mouse brain, termed NMO-IgG, was discovered in patients with this condition and soon determined to target aquaporin-4 (28, 29). While tissue indirect immunofluorescence (TIIF) was initially used to detect AQP4-IgG, evaluations of alternative test methodologies followed rapidly. Immunoprecipitation assays were reported to moderately enhance sensitivity particularly when combined with TIIF, although occasional false-positives were described in patients without characteristic TIIF staining (1, 30). Evaluations of enzyme-linked immunosorbent assay (ELISA) also suggested higher overall sensitivity than TIIF (31). However, the possibility for false-positive results using ELISA was also reported, highlighting the need for more sensitive and specific assays to detect AQP4-IgG (32).

### The Emergence of Highly Sensitive and Specific AQP4-IgG Cell-Based Assays

Cell-based assays (CBAs) using HEK cells transfected with AQP4 were shown to have high sensitivity and specificity, although initially their restriction to specialized centers limited evaluation of their use in a high-thoroughput laboratory setting (1, 30, 33). The advent of commercially available AQP4-IgG ELISA and CBA led to increased test accessibility across clinical service laboratories, and created the need for comparative studies evaluating their diagnostic performance. A multicenter comparison study found that CBAs detecting AQP4-IgG by quantitative flow cytometry (FACS) or visual observation of immunofluorescence (IF) had excellent specificity and the highest sensitivity for NMOSD when compared to other assays (34). AQP4-IgG ELISA with lowered cut-off values was reported to have high sensitivity but at some expense to specificity, suggesting the need for confirmatory testing by CBA in patients with low ELISA values (34). Meanwhile, both TIIF and immunoprecipitation assays were found to have suboptimal sensitivity for AQP4-IgG, limiting their usefulness in screening patients with suspected NMOSD for this antibody (34). These findings indicated that CBAs are the preferred methodology when testing for AQP4-IgG, a conclusion supported by a subsequent systematic review of AQP4-IgG assays (35).

### Variations Across AQP4-IgG CBAs: Comparisons of AQP4 Isoforms, Use of Live or Fixed Cells, and CBA-FACS or CBA-IF

Several studies have investigated potential differences in variations across CBAs, including the choice of transfected AQP4 isoform, use of live or fixed cells, and use of CBA-FACS or CBA-IF. Human AQP4 exists as two major isoforms that differ in their N termini, the M1 isoform and the M23 isoform (36). It has been debated whether CBAs using the shorter M23 isoform might have higher sensitivity (37, 38). However, a study comparing a widely used commercial M1-based CBA to a newly developed M23-based CBA found no significant differences in test performance, similar to the observation by another group that increased signal of an M23-based CBA was offset by an increase in background fluorescence (35, 39). A multicenter comparison of AQP4-IgG assays that included in-house live M23 CBAs suggested these assays had slightly higher sensitivity, but with rare-false positives reported (40). Another study that included comparison of in-house live M1 and M23 CBAs found that the M1 CBA was preferable in a clinical laboratory service setting, due to false-positive results using the M23 CBA (41). Overall, it would seem that M1 CBAs are suitable for routine clinical use; while there may be potential for increased sensitivity by using M23 CBAs, care should be taken to ensure this does not come at the expense of specificity if developed for clinical service implementation.

With regard to live vs. fixed CBAs for AQP4-IgG detection, an early study reported a slight reduction in sensitivity of a commercial AQP4-IgG assay using formaldehyde-fixed HEK cells when compared to live HEK cells in a small cohort of patients, which the authors posited might be related to epitope alteration due to fixation (34, 35). However, a more recent study that incorporated the 2015 international consensus diagnostic criteria for NMOSD into its comparison of AQP4-IgG assays found excellent specificity and a comparably high sensitivity of both fixed and live CBAs, and confirmed superiority of CBAs over ELISA and TIIF (42). The findings of this study indicate that commercial fixed CBA is a viable option for clinical laboratories that are interested in offering AQP4-IgG testing but do not have the capability to perform live CBA, which is relatively time-consuming and labor-intensive. Among specialized laboratories that offer live CBA, both CBA-IF and CBA-FACS are in use. A multicenter study that included both live CBA-IF and CBA-FACS assays found, somewhat surprisingly, that live CBA-FACS assays varied substantially in terms of sensitivity and specificity, highlighting the importance of live CBA-FACS optimization prior to clinical implementation (40). This is exemplified by one center that, after rigorously evaluating live CBA-FACS compared to other AQP4-IgG assays and determining their live M1 CBA-FACS assay to perform optimally, subsequently demonstrated high sensitivity and exceptional specificity of their assay without false-positives in a high throughput setting (41, 43).

### Serum vs. CSF Testing for AQP4-IgG

While the majority of early investigations into AQP4-IgG were based on serum testing alone, reports emerged of CSF positivity in seronegative patients with NMOSD. One study described three patients with compatible disease phenotypes and NMO-IgG positivity by TIIF that was restricted to CSF; however, the significance of this finding was unclear, given the potential for increased non-specific background staining of serum TIIF that may impede visualization of NMO-IgG staining (44). A study using CBA to detect AQP4-IgG found that only 8/20 patients with serum AQP4-IgG positivity were also positive in CSF, indicating that serum is more sensitive than CSF (45). Another comparison study of serum and CSF AQP4-IgG positivity using CBA found that CSF was positive in only 21/31 patients with AQP4-IgG seropositive NMOSD and none of 14 patients with AQP4-IgG seronegative NMOSD, thereby providing additional evidence that the sensitivity of serum is higher than CSF (46). This was further corroborated by another study evaluating serum vs. CSF testing for AQP4-IgG by CBA as part of clinical service evaluation, in which serum was more sensitive than CSF and no additional cases were detected by CSF testing (47). High serum AQP4-IgG titers have consistently been found to predict CSF positivity, suggesting a relative lack of intrathecal synthesis and need for a critical serum/CSF gradient before AQP4-IgG is detectable in CSF (45–47). Given these findings, serum is recommended over CSF when submitting samples to test for AQP4-IgG by CBA.

### Ensuring the Use of AQP4-IgG CBAs in Clinical Practice

Overall, it would seem that any marginal differences in the performance of various AQP4-IgG CBAs reported in the literature, such as those using the M1 vs. M23 isoform or live vs. fixed cells, has less relevance to clinical practice than the overall superior diagnostic performance of CBAs when compared to other test methodologies. Raising awareness of this among both clinicians and laboratorians is critical because assays that are liable to generate false-positive results, such as AQP4-IgG ELISA, are still commonly ordered for patients with suspected NMOSD and can lead to misdiagnosis (48, 49). Such assays should be phased out of clinical practice wherever possible, because accessibility to CBA is now widespread due to commercial assays using fixed cells that perform comparably well to live CBAs offered at specialized centers. Given that AQP4-IgG serostatus now features prominently in diagnostic criteria and clinical trials of drugs for NMOSD, accurate detection of this antibody has become especially important to patient diagnosis and treatment (10, 50).

## Antibodies Against Myelin Oligodendrocyte Glycoprotein

### The Early Days of MOG-IgG Detected by Western Blot and ELISA: An Antibody of Unclear Significance in Multiple Sclerosis

Myelin oligodendrocyte glycoprotein (MOG) is membrane protein specific to the central nervous system that is found predominantly on the surface of myelin sheaths (51). A study of MOG-IgG detected by western blot in patients with clinical isolated syndrome found that antibody positivity was a significant predictor of early conversion to clinically definite MS, but this finding was not reproducible in another study using the same western blot protocol (52, 53). These contradictory data are representative of much of the conflicting early works investigating MOG-IgG detected by immunoblots or ELISAs that use denatured protein as the substrate, which have been summarized previously (11). The lack of consistency across studies using these assays, coupled with their high positivity rate among healthy controls, has been attributed to the detection of antibodies against non-native MOG epitopes (11).

### Antibodies Against Natively-Folded MOG Detected by CBA: A Biomarker of a Distinct Non-MS Inflammatory Demyelinating Disease

The development of an assay to detect antibodies against natively-folded MOG was a breakthrough that led to the determination of MOG-IgG positivity in approximately 20% of patients with acute disseminated encephalomyelitis (ADEM), compared to only 1% of patients from North America with adult-onset MS (54). Using live CBAs expressing conformational MOG it was subsequently demonstrated that a minority of patients with AQP4-IgG seronegative NMOSD were MOG-IgG positive, expanding the clinical phenotype of this antibody beyond ADEM to include optic neuritis and myelitis (55–57). It has been found that live CBAs expressing full-length human MOG α1 isoform enable detection of MOG-IgG with high sensitivity, while use of secondary antibodies to IgG1 or IgG-Fcγ has been reported to improve specificity (6, 58, 59). Reports of differential IgG binding patterns to MOG isoforms, as well as of reactivities of MOG-IgG subclasses other than IgG1, are of interest and would benefit from further study (59–61). Such future studies could facilitate the refinement of CBAs used to detect MOG-IgG, which have helped to define MOG-antibody-associated disease (MOGAD) as an entity distinct from MS (62–64).

### Comparisons of Live and Fixed MOG-IgG CBAs

In addition to live CBAs implemented at specialized centers, fixed CBA for MOG-IgG detection has become commercially available. This has spurred comparative studies of live and fixed CBAs for MOG-IgG, which can serve to inform the utility of these assays in clinical laboratories. A multicenter case-control study of three different MOG-IgG CBAs (two live CBAs and one fixed CBA) reported an overall high degree of agreement across assays, although the specificity of fixed CBA (98%) was slightly lower than both live CBAs (>99%) (65). The sensitivity of CBAs in this study, which defined any patient with a compatible MOGAD phenotype as a true-positive, ranged from 23 to 28%, with sensitivity of fixed CBA falling between that of the two live CBAs. A subsequent study compared the reproducibility of 11 antibody assays, including seven live MOG-IgG CBAs and one fixed MOG-IgG CBA (66). The overall concordance of these eight CBAs was 90% when analyzing clearly positive or negative samples, which were classified as such by four testing centers using live CBA. This increased to 96% if the fixed CBA was excluded, which was driven by the exclusion of four false-negatives by fixed CBA (66). This would seem to suggest higher sensitivity of live CBA, although comparison to changes in overall concordance when excluding each live CBA was not reported. Taken together with the findings of the previous study, live CBAs for MOG-IgG likely have slightly higher diagnostic accuracy than fixed CBA, and could thus be useful to reconcile unexpected false-negative or false-positive results by fixed CBA. More striking, however, was that the overall concordance across all 8 CBAs when analyzing low-positive samples was only 33%, which is considerably lower than concordance across CBAs used to detect low levels of AQP4-IgG (40, 66). This greater variability in detection of low levels of MOG-IgG across both live and fixed CBAs calls into question whether these low levels can reliably distinguish MOGAD from other disease processes (discussed later on), and represents a barrier to patient disease classification for the purposes of sensitivity/specificity analyses in diagnostic accuracy studies. The development of international consensus criteria for MOGAD that are independent of the antibody result would be helpful to analyze patients with discordant low-positive MOG-IgG across CBAs, and could be incorporated into future comparative studies of assay performance like has been done with AQP4-IgG testing for NMOSD (42).

### Serum vs. CSF Testing for MOG-IgG

Similar to AQP4-IgG, an early investigation of serum vs. CSF testing of MOG-IgG by CBA found a relative lack of intrathecal synthesis and indicated that serum was the preferred sample to test (67). In this study only 12/18 seropositive MOG-IgG patients also had MOG-IgG detected in CSF, although seronegative MOG-IgG patients with a compatible MOGAD phenotype did not undergo CSF MOG-IgG testing to assess for potential identification of additional cases (67). Another study identified three patients with non-MS demyelinating disease that were seronegative for MOG-IgG but found to be positive in CSF using CBA (68). This led the authors to hypothesize that, while overall sensitivity of serum testing for MOG-IgG was higher, CSF testing might identify some additional cases of MOGAD (68). This hypothesis was supported by autopsy findings of a patient with rapidly progressive meningoencephalomyelitis and MOG-IgG positivity in CSF only, which showed similar neuropathological findings to that described in seropositive MOG-IgG cases (69). A recent comparative study determined that 11/38 patients with MOGAD had CSF positivity only compared to 0/36 patients with AQP4-IgG-positive NMOSD, with a significantly higher proportion of patients with MOGAD found to have evidence of intrathecal synthesis of MOG-IgG (70). This was corroborated by another study that identified nine patients with non-MS inflammatory demyelinating diseases compatible with MOGAD and isolated CSF MOG-IgG positivity, eight of whom were found to have intrathecal MOG-IgG synthesis (71). Importantly, however, four patients with MS and isolated CSF MOG-IgG positivity were also reported, suggesting the possibility of false-positive CSF results (71). To better determine the utility of serum vs. CSF MOG-IgG testing, a national laboratory analyzed consecutive samples referred for testing; among 118 patients with positivity in either fluid, isolated CSF MOG-IgG was only found in 4/118 and one had bacterial meningitis rather than MOGAD (72). Overall, these findings suggest that while a minority of patients with disease phenotypes consistent with MOGAD may exhibit isolated CSF MOG-IgG positivity, routine testing of CSF MOG-IgG in addition to serum is low-yield and the possibility of false-positives exists. Additionally, many CBAs used to test for MOG-IgG in serum are not currently clinically validated for detection of this antibody in CSF. For these reasons, it would seem reasonable at this time to limit CSF testing for MOG-IgG to patients in whom there is a high index of clinical suspicion and no more likely alternative diagnosis, pending further investigation of its diagnostic value.

### The Importance of Reviewing Titers When Interpreting Positive MOG-IgG Results, and the Question of Low-Titer MOG-IgG in MS

The need to consider antibody titer when interpreting positive MOG-IgG results was highlighted in a single-center study of consecutive patients tested for MOG-IgG by live CBA. This study found that the probability of a true-positive result varied significantly by titer, ranging from 100% with a titer of ≥1:1000 to only 51% in those with a titer of 1:20–1:40 (73). These findings indicate that antibody titer can help to gauge the likelihood of a true-positive result when encountering a positive MOG-IgG result in a patient with an atypical or equivocal phenotype for MOGAD, and should therefore be routinely reviewed as part of test interpretation. Importantly, titration may not be performed in clinical laboratories using fixed CBA for MOG-IgG detection, and cut-off titers for MOG-IgG seropositivity by live CBA can vary depending on factors such as the secondary antibody used (66); for these reasons, reporting of titers should be clarified with the testing laboratory to avoid misinterpretation.

One outstanding question is how to interpret MOG-IgG detected by CBA in a small portion of patients with MS, which is typically low-titer and has generally been classified as false-positive in diagnostic accuracy studies. This classification is supported by a literature review of patients with typical lesions for MS on magnetic resonance imaging (MRI) and MOG-IgG positivity by live or fixed CBA, who were found not to differ from what would be expected of clinically definite MS in terms of attack severity, treatment response, MRI evolution over time, and outcomes (74). However, it has been suggested that low titers of MOG-IgG in a patient with MS may indicate that they lie on a disease spectrum between the two entities, in part because of the high proportion of patients with MS among those classified as false-positives in studies of MOG-IgG (75). While it is true that low-titer MOG-IgG values classified as false-positives are often reported in patients with MS, this is confounded by the fact that MS is likely the most common alternative diagnosis among patients being tested for MOG-IgG in clinical practice; the greatest proportion of false-positives is thus expected to be patients with MS, even if no predilection for MOG-IgG detection in MS exists. To this point, a recent study that indiscriminately performed MOG-IgG testing in consecutive neurologic patients found that the majority with low antibody levels had non-inflammatory diseases such as stroke, rather than MS (76). Even if a higher proportion of low-titer MOG-IgG in MS compared to other diseases was systematically demonstrated, an additional challenge would be discerning whether low levels of MOG-IgG in this context contribute to immunopathogenesis or are an epiphenomenon of MS-related demyelination, i.e., whether the antibody is of clinical relevance or not; this adds a layer of complexity to both diagnostic accuracy studies and clinical practice that would benefit from dedicated evaluation.

## Conclusions

The discoveries of AQP4-IgG and MOG-IgG have transformed the evaluation of patients with suspected autoimmune myelopathy. Sensitive and specific CBAs to detect these antibodies have facilitated the diagnosis of AQP4-IgG-positive NMOSD and MOGAD in clinical practice, which are distinct disease entities. Serum is the preferred specimen for routine testing of both antibodies; isolated CSF MOG-IgG positivity in a minority of patients with compatible disease phenotypes, while intriguing, is a finding that requires further study. Fixed CBAs, which are relatively easy to implement in clinical laboratories and are thus now in widespread use, have dramatically improved test accessibility. For AQP4-IgG detection, fixed CBA has been found to perform comparably well to live CBA and should motivate replacement of other commercial assays that are still in use despite demonstrably inferior diagnostic performance, such as ELISA. Live CBAs seem to confer some diagnostic advantage over fixed CBAs for MOG-IgG detection, although future comparative studies would benefit from the development of international consensus diagnostic criteria for MOGAD that could aid in patient classifications for the purposes of sensitivity/specificity analyses. Such criteria could also help clinicians to reconcile possible clinically irrelevant or false-positive results that can occur with live or fixed MOG-IgG CBAs, generally at low titers and both in MS as well as various other diseases. In patients with atypical or equivocal phenotypes for MOGAD and a positive MOG-IgG result, discussion with the testing laboratory surrounding assay cut-offs as well as consideration of titer can help to avoid misdiagnosis.

## Author Contributions

This manuscript was entirely planned and drafted by SA and AB. Both authors contributed to the article and approved the submitted version.

## Funding

AB reports that he holds the London Health Sciences Centre and London Health Sciences Foundation Chair in Neural Antibody Testing for Neuro-Inflammatory Diseases.

## Conflict of Interest

The authors declare that the research was conducted in the absence of any commercial or financial relationships that could be construed as a potential conflict of interest.

## Publisher's Note

All claims expressed in this article are solely those of the authors and do not necessarily represent those of their affiliated organizations, or those of the publisher, the editors and the reviewers. Any product that may be evaluated in this article, or claim that may be made by its manufacturer, is not guaranteed or endorsed by the publisher.
